# Impact of macrophage differentiation on hematopoietic function enhancement by Shenzhu ErKang Syrup

**DOI:** 10.18632/aging.205358

**Published:** 2024-01-03

**Authors:** Yuan Li, Meng Teng, Hongxin Yang, Siyu Li, Xin Liu, Jicheng Zhang, Ye Qiu, Lanzhou Li

**Affiliations:** 1 Engineering Research Center of Chinese Ministry of Education for Edible and Medicinal Fungi, College of Plant Protection, Jilin Agricultural University, Changchun 130118, Jilin, China; 2 School of Life Sciences, Jilin University, Changchun 130012, Jilin, China; 3 School of Chemical and Biomolecular Engineering, Georgia Institute of Technology, Atlanta, GA 30332, USA; 4 Department of Pharmacy, Changchun University of Chinese Medicine, Changchun 130012, Jilin, China

**Keywords:** Shenzhu Erkang Syrup, hematopoietic function, intestinal flora, metabolomics, macrophages

## Abstract

Shenzhu Erkang Syrup (SZEK) is a traditional Chinese medicine that improves spleen and stomach function, tonifying the Qi and activating the blood; however, its therapeutic effects in hematopoietic dysfunction and their underlying mechanism remain unexplored. In this study, mice were given cyclophosphamide (100 mg/kg) by intraperitoneal injections for three days to produce hematopoietic dysfunction model. We investigated the hematopoietic effect and mechanism of SZEK in mice with hematopoietic dysfunction via histopathological examination, flow cytometry, enzyme-linked immunosorbent assay, and Western blotting combined with intestinal flora and serum metabolomics analysis. In mice with hematopoietic dysfunction, SZEK (gavage, 0.3 mL/25 g) alleviated pathological damage to the bone marrow and spleen; increased the number of naïve cells (Lin^−^), hematopoietic stem cells (Lin^−^Sca-1^+^c-Kit^+^), long-term self-renewing hematopoietic stem cells (Lin^−^Sca-1^+^c-Kit^+^CD48^−^CD150^+^), B lymphocytes (CD45^+^CD19^+^), and macrophages (CD11b^+^F4/80^+^) in the bone marrow; and reduced inflammation. Preliminary intestinal flora and serum metabolome analyses indicated that the pro-hematopoietic mechanism of SZEK was associated with macrophage differentiation. Further validation revealed that SZEK promoted hematopoiesis by decreasing the number of M2 macrophages and inhibiting the secretion of negative hematopoietic regulatory factors in mice with hematopoietic dysfunction.

## INTRODUCTION

Chemotherapy is a mainstay of clinical treatment for malignant tumors [1]. However, chemotherapeutic drugs, such as cyclophosphamide (CTX), paclitaxel, pemetrexed, and gemcitabine can cause bone marrow suppression [2], resulting in detrimental effects on the hematopoietic system [3]. Depression of the hematopoietic system can lead to anemia, hemorrhage, and infection [4]. Hematopoiesis involves the generation and differentiation of hematopoietic stem cells (HSCs), which can differentiate into long-term self-renewing HSCs (LT-HSCs), granulocytes, macrophages, B lymphocytes, T lymphocytes, and other hematopoiesis-associated cells [5]. Macrophages are crucial cells that influence HSC mobilization in the bone marrow and are essential for hematopoietic regulation [6]. Hematopoiesis occurs in hematopoietic organs, including the bone marrow and spleen, and is modulated by various cytokines such as interleukin (IL)-10 and transforming growth factor (TGF)-β [3, 7, 8]. Inflammation is thought to be a major regulator of HSC function, as widespread inflammation ages the bone marrow microenvironment and subsequently leads to hematopoietic depression [9, 10]. Bone marrow suppression in turn results in damage to hematopoietic organs, reduced number of hematopoietic cells, and disruption of inflammatory and hematopoietic-associated factors [3]. Consequently, there is an urgent need for adjuvant drugs that can safely and effectively protect against hematopoietic damage to mitigate the severe adverse effects of chemotherapy.

Iron, granulocyte colony-stimulating factor, recombinant human erythropoietin, and chemosynthetic myeloprotective agents are commonly used to alleviate hematopoietic damage. However, such treatments require frequent repetition and are associated with substantial side effects [11]. Chinese medicines have been extensively studied to alleviate chemotherapy-induced hematopoietic dysfunction. Chinese medicine is characterized by its strong pharmacological effects and low cytotoxicity, and exerts therapeutic effects by regulating homeostasis, resulting in a calm therapeutic effect [12]. Danggui Buxue Decoction can reduce chemotherapy-induced myelosuppression and has been widely used in clinical cancer treatment [13]. Shenzhu ErKang Syrup (SZEK) is a traditional Chinese medicine for children that is composed of *Atractylodes macrocephala* rhizome stir-fried with bran, *Poria cocos* (Schw.) Wolf, *Crataegus pinnatifida* Bunge, Radix astragali prepaprata, *pseudostellaria radix*, bee yellow, Radix polygoni multiflori prepaprata, *Dioscorea polystachya* Turczaninow, *Angelica sinensis* (Oliv.) Diels, *Platycodon grandiflorus* (Jacq.) A. DC., orange peel, Polygala *tenuifolia* Willd., *Glycyrrhiza uralensis* Fisch., *DolichoslablabL.*, Massa medicata fermentata, and colored malt. The marketing approval for SZEK has been obtained in China, where it is utilized to strengthen spleen and stomach function, tonifying the Qi and activating the blood. However, the therapeutic effects and mechanisms of action of SZEK in hematopoietic dysfunction have not been systematically investigated.

The intestinal flora can influence host hematopoietic system either through a direct effect on hematopoietic stem and precursor cells or occur indirectly through their detection by niche supporting cells [14]. Studies have confirmed that the intestinal flora can influence and support hematopoiesis by absorbing dietary nutrients [15], and disturbances in the intestinal flora can lead to metabolic disorders and disease episodes in hosts. Chinese medicines can modulate the composition of intestinal flora by selectively inhibiting or promoting the growth of intestinal microorganisms. Intestinal flora metabolizes these medicines, potentially enhancing their effectiveness or reducing their toxicity [16]. For example, Rhubarb Peony decoction can alter the composition of the intestinal flora by enriching beneficial bacteria and suppressing pathogenic bacteria to restore Th17/Treg homeostasis and reduce the level of pro-inflammatory cytokines, such as IL-6 and tumor necrosis factor (TNF)-α [17], and intestinal flora affects the hematopoietic regulatory efficacy of Danggui Buxue Tang by affecting the plasma concentrations of active ingredients [18]. Alterations in the composition of the intestinal flora can affect the metabolic phenotypes that function as gut microbial messengers, thereby influencing host inflammation and further affecting the hematopoietic system [19]. The intestinal flora and serum differential metabolites may be closely correlated, and drugs can affect the hematological system of the host through the mutual regulation of these two factors. For example, Taohong Siwu Decoction can improve blood deficiency and blood stasis syndrome in rats by targeting intestinal flora and metabolomics [20]. Therefore, integrating analyses of intestinal flora and serum metabolomics may enable a more comprehensive analysis of the regulatory effects of medicines on hematopoietic function and the associated mechanisms.

In this study, we established a mouse model of hematopoietic dysfunction via intraperitoneal CTX injection, explored the hematopoietic improvement effect of SZEK in mice, and studied the mechanism of action of SZEK in promoting hematopoiesis by combining microbial 16S rRNA high-throughput sequencing technology with serum metabolomic analysis based on ultra-high-performance liquid chromatography-mass spectrometry.

## RESULTS

### Effect of SZEK on histopathology in hematopoietically dysfunctional mice

**Figure 1:**
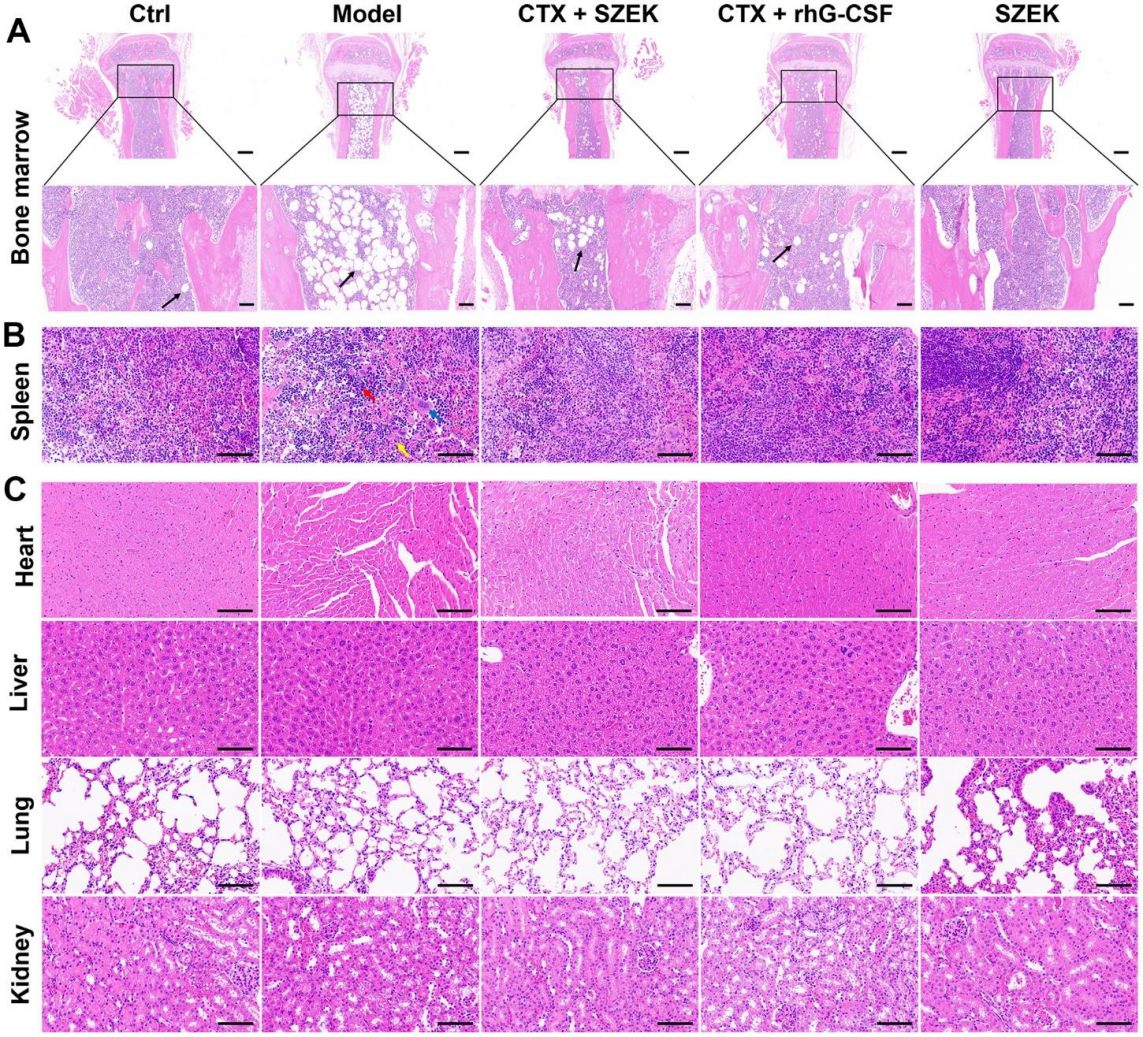
**SZEK improves histopathology in mice with hematopoietic dysfunction.** SZEK restores histomorphology in (**A**) bone marrow (5×, scale bar: 200 μm; 20×, scale bar: 50 μm) and (**B**) spleen (200×, scale bar: 100 μm) of mice with hematopoietic dysfunction without affecting the tissue morphology of (**C**) heart, liver, lung, and kidney (200×, scale bar: 100 μm). The black arrow shows myeloid cell adipocytosis in **A**. In **B**, lymphocytes are denoted by red arrows, multinucleated macrophages are denoted by blue arrows, and neutrophils are denoted by yellow arrows. Ctrl, control; Model, hematopoietic dysfunction model.

The bone marrow and spleen are active hematopoietic tissues in mice [21]. Histopathological changes in the bone marrow and spleen tissues were confirmed via hemeatoxylin and eosin (H&E) staining. Compared with normal mice, the Model group of mice administered CTX exhibited a highly abnormal bone tissue structure, with a prominent reduction in the number of bone trabeculae in the bone marrow cavity and heavy steatosis of the bone marrow cells. This resulted in the generation of a large number of adipocytes and consequently a reduction in the number of bone marrow cells, which was ameliorated by the administration of SZEK and recombinant human granulocyte colony-stimulating factor (rhG-CSF) (Figure 1A). Furthermore, SZEK and rhG-CSF alleviated the structural disorganization of splenic nodules, considerably reduced the number of lymphocytes, and substantially increased the number of multinucleated macrophages as well as neutrophil infiltration in the spleen of mice with hematopoietic dysfunction (Figure 1B). When administered alone, SZEK did not affect the bone marrow or spleen of the mice (Figure 1A, 1B), and no significant changes were observed in the heart, liver, lung, or kidney of any group (Figure 1C). This demonstrates the protective effect of SZEK in curing CTX-induced bone marrow and spleen injuries.

### Effect of SZEK on hematopoietic cells in bone marrow of mice with hematopoietic dysfunction

**Figure 2:**
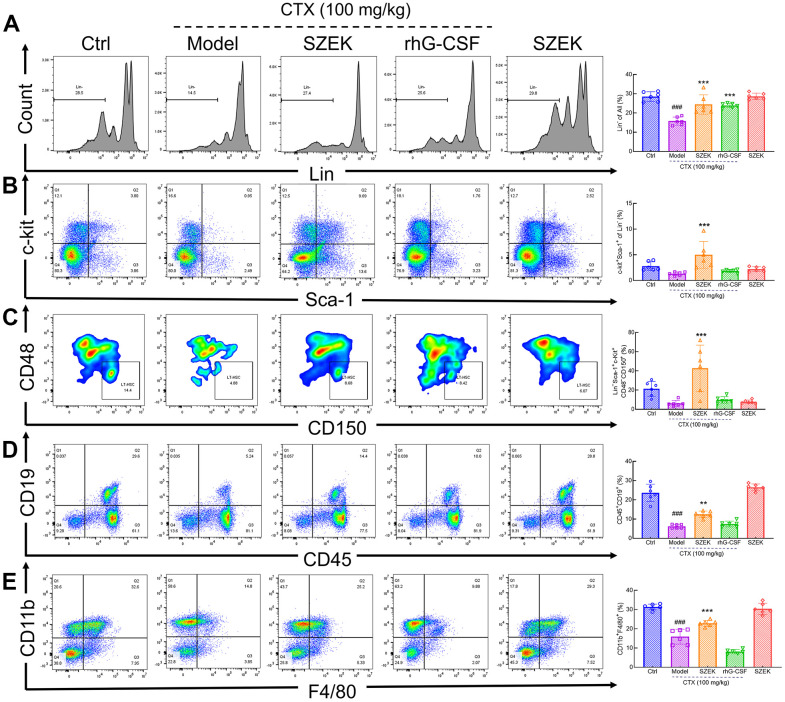
**SZEK regulates hematopoietic cell levels in the bone marrow of mice with hematopoietic dysfunction.** SZEK increased the number of (**A**) naïve cells (Lin^−^), (**B**) HSCs (Lin^−^Sca-1^+^c-Kit^+^), (**C**) LT-HSCs (Lin^−^Sca-1^+^c-Kit^+^CD48^−^CD150^+^), (**D**) B lymphocytes (CD45^+^CD19^+^) and (**E**) macrophages (CD11b^+^F4/80^+^) in bone marrow of mice with hematopoietic dysfunction (n=6, ^###^ *P*<0.001 versus Ctrl. ^**^ *P*<0.01, and ^***^ *P*<0.001 versus Model). Ctrl, control; Model, hematopoietic dysfunction model.

The number of hematopoietic-associated cells in the bone marrow is one of the indicators that directly evaluates the hematopoietic function of the organism [4, 22]. CTX had an inhibitory effect on the myeloid population including naïve cells (Lin^−^), HSCs (Lin^−^Sca-1^+^c-Kit^+^), LT-HSCs (Lin^−^Sca-1^+^c-Kit^+^CD48^−^CD150^+^), B lymphocytes (CD45^+^CD19^+^) and macrophages (CD11b^+^F4/80^+^) compared with normal mice (Figure 2A–2E). Following SZEK administration, the numbers of naïve cells, HSCs, LT-HSCs, B lymphocytes, and macrophages significantly increased by 54.97% (*P*<0.001), 279.57% (*P*<0.001), 597.90% (*P*<0.001), 100.00% (*P*<0.01), and 43.66% (*P*<0.001) (Figure 2A–2E), respectively, in bone marrow of mice with hematopoietic dysfunction, suggesting that SZEK can enhance hematopoiesis in mice by promoting the proliferation of bone marrow hematopoietic cells.

### Effect of SZEK on inflammatory factors in spleen and serum of mice with hematopoietic dysfunction

**Figure 3:**
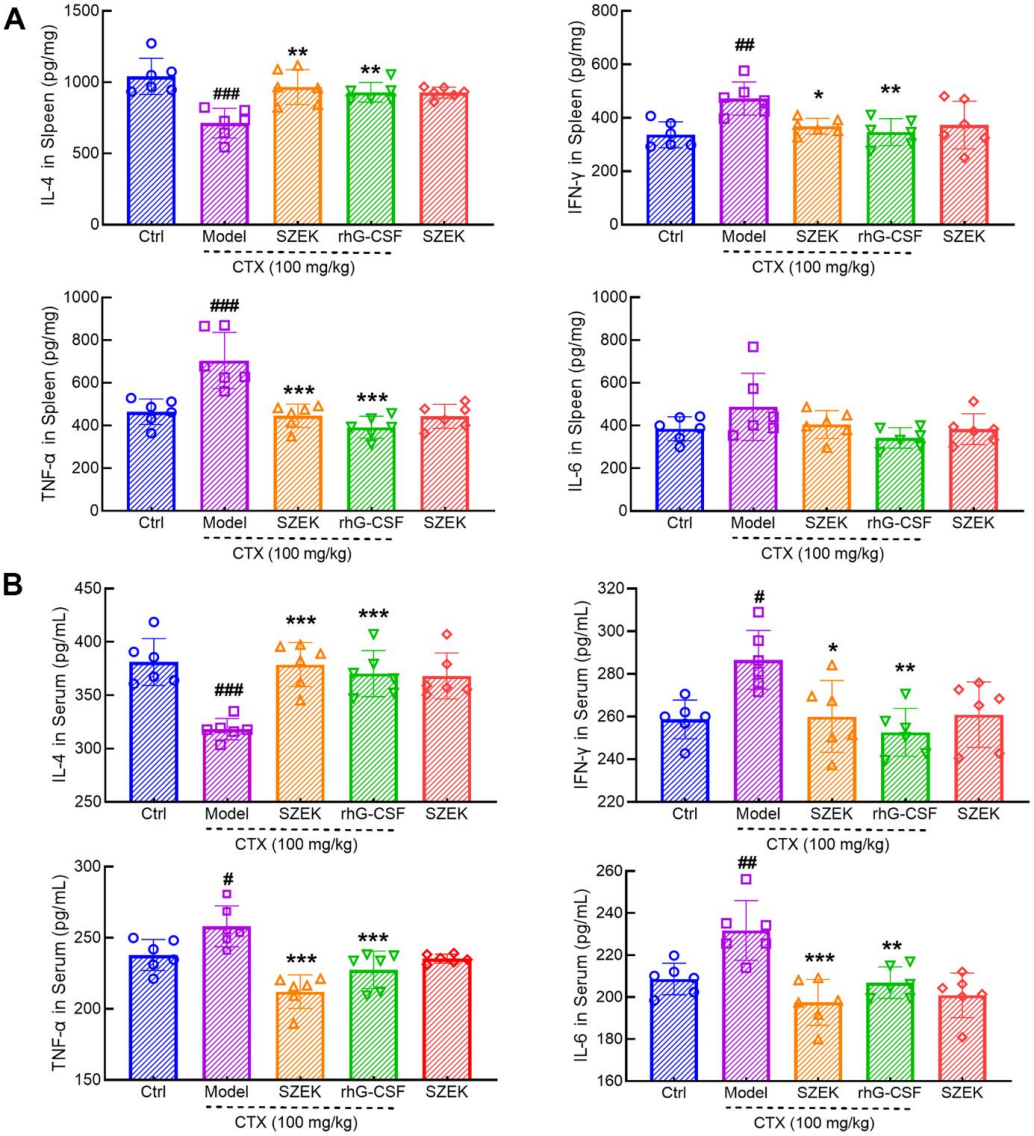
**SZEK regulates cytokine expression in mice with hematopoietic dysfunction.** SZEK increases the expression of IL-4 and decreased the expression of IFN-γ, TNF-α, and IL-6 in (**A**) spleen and (**B**) serum of mice with hematopoietic dysfunction (n=6, ^#^ *P*<0.05, ^##^ *P*<0.01 and ^###^ *P*<0.001 versus Ctrl. ^*^ *P*<0.05, ^**^ *P*<0.01, and ^***^ *P*<0.001 versus Model). Ctrl, control; Model, hematopoietic dysfunction model.

Inflammatory signaling regulates the development and homeostasis of HSCs and hematopoietic progenitor cells (HPCs) and is critical for the hematopoietic system [23]. In mice with hematopoietic dysfunction, the levels of IL-4 in the spleen were significantly decreased by 31.55% (*P*<0.001) whereas that of interferon (IFN)-γ, TNF-α, and IL-6 was increased by 40.29% (*P*<0.01), 51.62% (*P*<0.001), and 26.56%, respectively (Figure 3A). SZEK treatment increased IL-4 levels by 35.60% (*P*<0.01), and decreased IFN-γ, TNF-α, and IL-6 levels by 21.96% (*P*<0.05), 36.74% (*P*<0.001), and 16.96%, respectively, in the spleen of mice with hematopoietic dysfunction (Figure 3A). Similarly, a significant decrease in IL-4 by 16.53% (*P*<0.001) and increases in IFN-γ, TNF-α, and IL-6 levels by 10.75% (*P*<0.05), 8.49% (*P*<0.05), and 11.08% (*P*<0.01), respectively, were observed in the serum of mice with hematopoietic dysfunction (Figure 3B). Following SZEK treatment, serum IL-4 levels increased by 19.02% (*P*<0.001) and serum IFN-γ, TNF-α, and IL-6 levels decreased by 9.24%, 17.80%, and 14.80%, respectively (*P*<0.05, Figure 3B). The results of enzyme-linked immunosorbent assay (ELISA) indicate that SZEK can reduce inflammation in the spleen and serum of mice with hematopoietic dysfunction.

### Effect of SZEK on intestinal flora of mice with hematopoietic dysfunction

**Figure 4:**
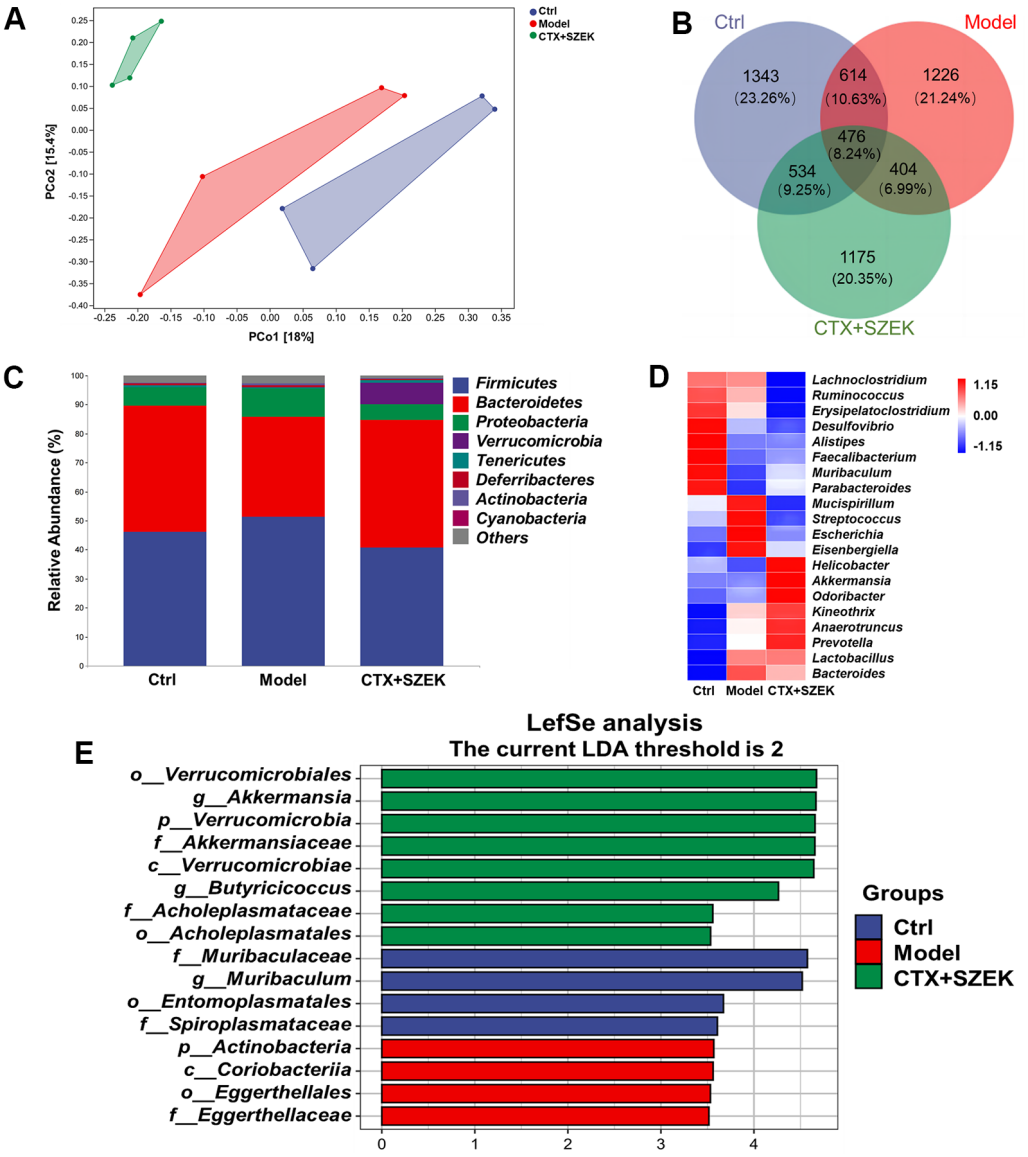
**SZEK alters intestinal flora in mice with hematopoietic dysfunction (n=4).** (**A**) Distance matrix and principal coordinate analysis of beta diversity. (**B**) Venn diagram. (**C**) Relative abundance analysis of intestinal flora between groups at the phylum. (**D**) Heat map of top 20 species composition at genus level. (**E**) LEfSe analysis of altered gut bacteria. Ctrl, control; Model, hematopoietic dysfunction model.

Recent studies have demonstrated that the intestinal flora can affect hematopoiesis [15]. Beta diversity analyses based on Bray–Curtis and principal coordinate analysis revealed differences among the Ctrl, Model, and SZEK groups, indicating distinct differences in their community profiles (Figure 4A). Common and unique operational taxonomic unit (OTUs) of the Ctrl, Model, and SZEK groups were analyzed, and the groups were found to have 1343, 1226, and 1175 unique OTUs, respectively, and 476 common OTUs (Figure 4B). Taxonomic analysis at the phylum level revealed that four differential bacteria were downregulated in the Model group, including Bacteroidetes and Verrucomicrobia, and four were upregulated in the Model group, including Firmicutes, Proteobacteria, and Actinobacteria. However, most of these alterations were recovered after SZEK administration (Figure 4C and Supplementary Table 1). At the genus level, the abundances of bacteria, such as *Faecalibacterium* and *Akkermansia*, were decreased in mice with hematopoietic dysfunction, while the abundances of *Streptococcus* increased. After SZEK administration, the abundances of bacteria such as *Faecalibacterium*, *Akkermansia*, *Parabacteroides*, and *Prevotella* increased, whereas those of bacteria such as *Erysipelatoclostridium* and *Streptococcus* decreased (Figure 4D and Supplementary Table 2). The biomarker bacteria in each group were analyzed using Linear discriminant analysis Effect Size (LEfSe) in combination with Linear Discriminant Analysis (LDA), and four markers in the Ctrl group, four markers in the Model group, and eight markers, including genus *Akkermansia*, phylum Verrucomicrobia, and families Akkermansiaceae and Verrucomicrobiae in the SZEK group were identified (Figure 4E and Supplementary Table 3). The results confirm that SZEK can exert a regulatory effect on the intestinal flora of mice with hematopoietic dysfunction.

### Effect of SZEK on serum metabolites in mice with hematopoietic dysfunction

**Figure 5:**
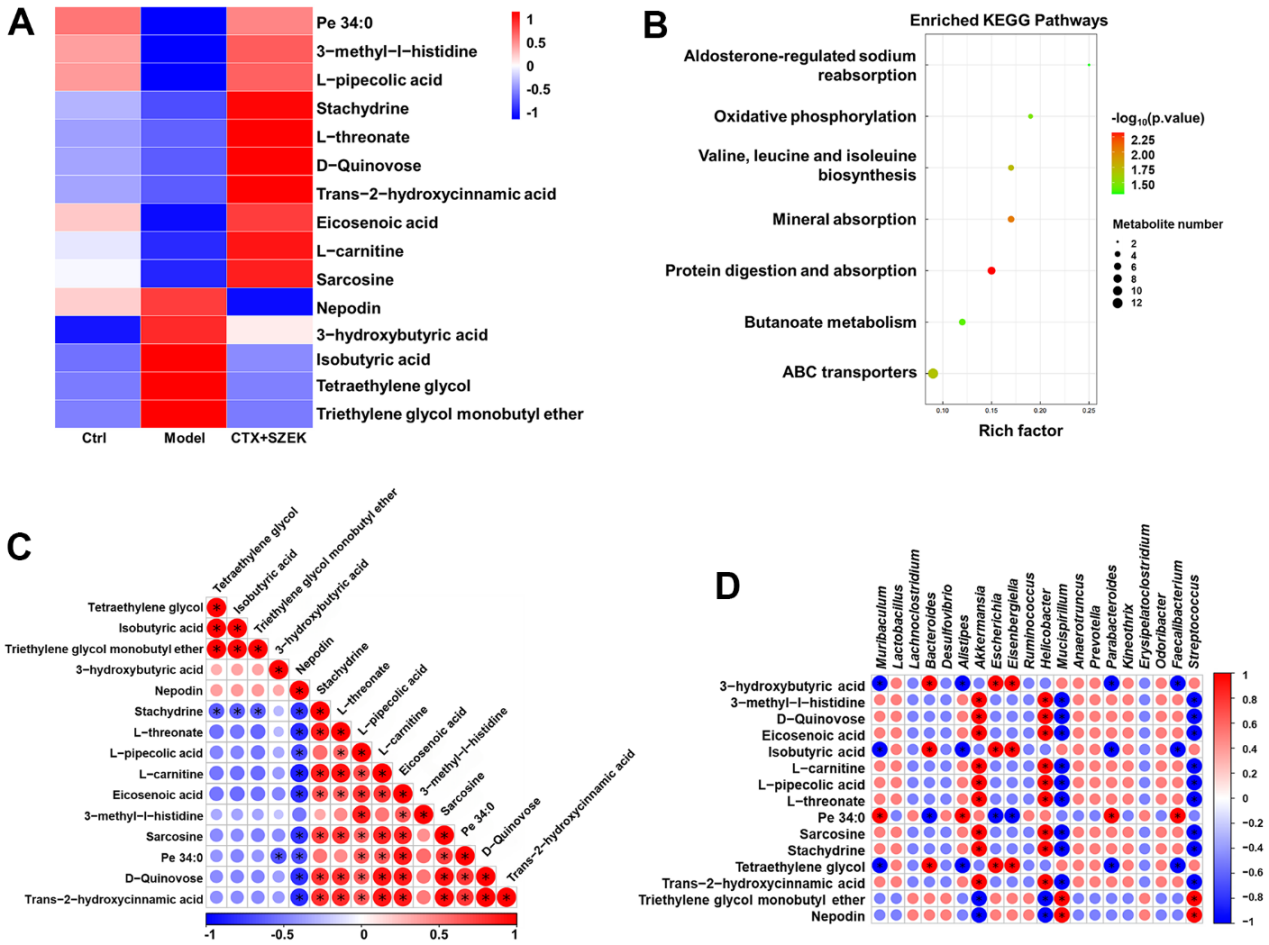
**SZEK affects serum metabolite levels in mice with hematopoietic dysfunction (n=4).** (**A**) Heat map analysis of differential metabolites. (**B**) KEGG pathways analysis of differential metabolites. (**C**) Correlative heat map analysis between differential metabolites. (**D**) Correlative heat map analysis between differential metabolites and intestinal flora. Ctrl, control; Model, hematopoietic dysfunction model.

Untargeted metabolomics facilitates the comprehensive and systematic analysis of metabolic profiles and identification of differential metabolites to explore the mechanisms of hematopoietic regulation based on metabolic function [24]. Substances satisfying both Orthogonal Partial Least Squares Discrimination Analysis VIP>1 and *P*<0.05 were defined as significantly different metabolites, and the top 15 significantly different metabolites were selected for subsequent bioconfidence analysis, including cluster, pathway, and correlation analyses (Figure 5A–5D). Compared to normal mice, the serum levels of 10 metabolites, including trans-2-hydroxycinnamic acid (T2HCA), L-threonate, L-carnitine, stachydrine, L-pipecolic acid, sarcosine, and 3-methyl-l-histidine (3-MEH), decreased, and those of five metabolites, including nepodin, 3-hydroxybutyric acid (3-HBA), and isobutyric acid, increased in model mice, which were reversed by SZEK administration (Figure 5A and Supplementary Table 4). Metabolic and signaling pathways significantly affected by differential metabolites were identified by Fisher's exact test-based Kyoto Encyclopedia of Genes and Genomes (KEGG) pathway enrichment analysis, and metabolic pathways found to be involved in these metabolites included aldosterone-regulated sodium reabsorption; oxidative phosphorylation; valine, leucine, and isoleucine biosynthesis; mineral absorption; protein digestion and absorption; butanoate metabolism; and ATP-binding cassette transporter (Figure 5B). Analysis of correlations between significantly different metabolites revealed positive correlations between T2HCA, L-threonate, L-carnitine, stachydrine, L-pipecolic acid, sarcosine, and 3-MEH, which were upregulated after SZEK administration, as well as between nepodin, 3-HBA, and isobutyric acid, which were downregulated metabolites, whereas negative correlations were observed between the upregulated and downregulated metabolites (Figure 5C). Correlations between differential flora and significantly altered metabolites were analyzed, and positive correlations were found between beneficial bacteria such as *Akkermansia* and *Prevotella* and beneficial metabolites such as L-threonate, L-carnitine, L-pipecolic, sarcosine, stachydrine, and T2HCA. Negative correlations were found between pathogenic bacteria, such as *Erysipelatoclostridium*, and these beneficial metabolites (Figure 5D). These results suggest that SZEK may alter the composition of the intestinal flora and further affect serum metabolites and hematopoiesis.

### Effect of SZEK on macrophage differentiation in mice with hematopoietic dysfunction

**Figure 6:**
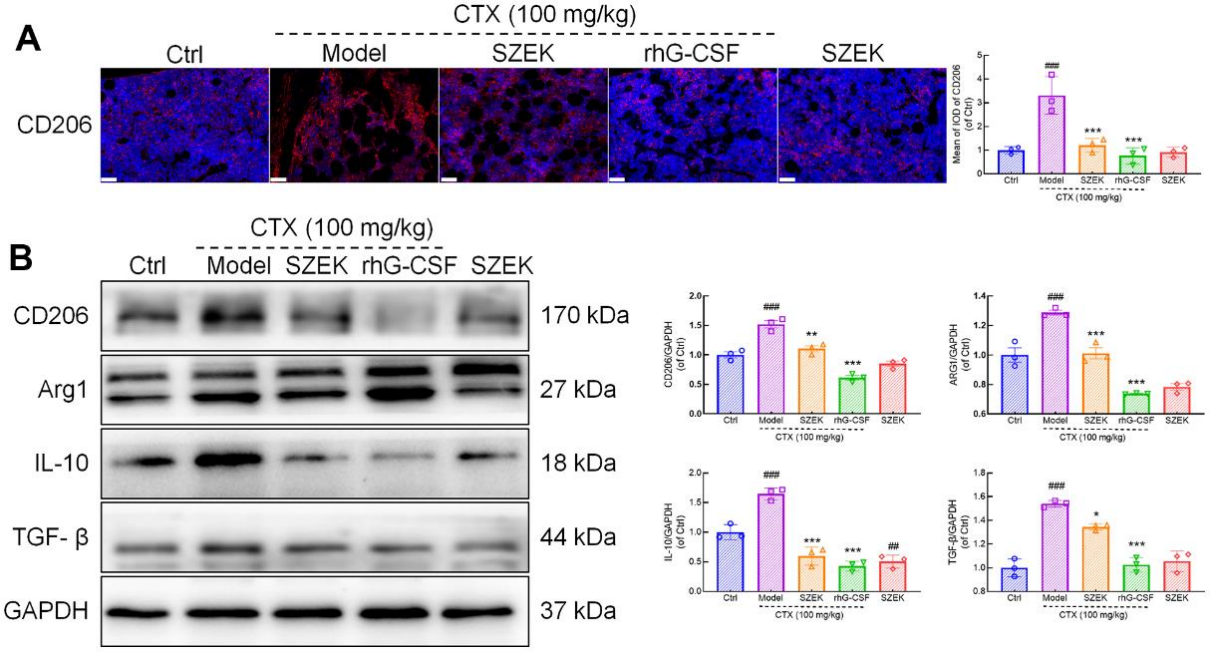
**SZEK inhibits M2 macrophage polarization and protein expression in spleen.** (**A**) Immunofluorescence analysis of CD206 in bone marrow of mice with hematopoietic dysfunction (200×, scale bar: 50 μm). (**B**) Expression levels of CD206, ARG1, IL-10, and TGF-β in spleen of mice with hematopoietic dysfunction detected by Western blotting. (n=3, ^###^ *P*<0.001 versus Ctrl. ^*^ *P*<0.05, ^**^ *P*<0.01, and ^***^ *P*<0.001 versus Model). Ctrl, control; Model, hematopoietic dysfunction model.

Macrophages play a key role in hematopoiesis [6], and are typically categorized into two types, M1 and M2, based on their activation status [25]. Among the results of intestinal flora and metabolome, some intestinal bacteria and metabolites were found to be able to influence macrophage differentiation and activity. The relationship between the pro-hematopoietic action of SZEK and macrophage regulation was further explored. CD206 and arginase-1 (ARG1) are markers of M2 macrophages [26]. Immunofluorescence showed that the expression of CD206 in the bone marrow of mice with hematopoietic dysfunction increased by 230.89% (*P*<0.001) compared to that in normal mice, and decreased by 63.52% (*P*<0.001) and 77.04% (*P*<0.001) after SZEK or rhG-CSF administration, respectively (Figure 6A). Similarly, in the spleen of mice with hematopoietic dysfunction, the protein levels of CD206 and ARG1 increased by 51.88% (*P*<0.001) and 28.71% (*P*<0.001), respectively, compared to the Ctrl group; SZEK administration reduced CD206 and ARG1 proteins by 27.34% (*P*<0.01) and 21.44% (*P*<0.001), respectively (Figure 6B). IL-10 and TGF-β are cytokines associated with M2 macrophages [27]. Compared to the Ctrl group, IL-10 levels were increased by 64.69% (*P*<0.001) and TGF-β levels were increased by 54.08% (*P*<0.001) in the spleen of mice with hematopoietic dysfunction, and after SZEK administration, they were reduced by 63.79% (*P*<0.001) and 12.81% (*P*<0.05), respectively (Figure 6B). These data suggest that SZEK can inhibit the levels of M2 macrophages and related cytokines in mice with hematopoietic dysfunction.

## DISCUSSION

Chemotherapy is one of the main treatment methods for cancer; however, the treatment process leads to bone marrow suppression and impair hematopoiesis [28]. SZEK is used as an herbal medicine for children with loss of appetite, restless sleep, excessive sweating, malnutrition, and anemia due to weakness of the spleen and stomach. The components of SZEK, such as, Radix polygoni multiflori prepaprata, *Angelica sinensis* (Oliv.) Diels, and Radix polygoni multiflori prepaprata, have effects on promoting hematopoiesis [29, 30]. This study confirmed the protective effects of SZEK in the bone marrow and spleen of mice with hematopoietic dysfunction. The bone marrow is the primary site of hematopoiesis [31]. It contains a unique microenvironment that provides niches that support self-renewal and differentiation of HSC, multipotent progenitors, and lineage committed progenitors to produce the large number of blood cells required to sustain life [32]. And the spleen is an important blood-forming organ [33]. HSCs reside in both bone marrow and spleen throughout the life of a mouse [34]. SZEK administration attenuated the pathological damage caused by CTX in the bone marrow and spleen, alleviated discrete bone marrow cells, and reduced inflammation in the spleen.

HSCs residing in the bone marrow are essential for blood production and hematopoiesis [35]. In mice, HSCs that give rise to all blood cell lineages are defined as a Lin^-^, c-Kit^+^, Sca-1^+^ (HSCs) populations, and can be further derived into various hematopoietic and functional blood cells, including LT-HSCs, B lymphocytes, and macrophages [36, 37]. SZEK administration increased the numbers of naïve cells, HSCs, LT-HSCs, B lymphocytes, and macrophages in the bone marrow of hematopoietically impaired mice, reflecting an improved effect on hematopoiesis. Inflammatory factors regulate HSC development, including emergence, trafficking, and differentiation [38], and widespread inflammation can lead to impaired hematopoiesis [10]. The cytokine IL-4 exhibits anti-inflammatory properties, whereas IFN-γ, TNF-α, and IL-6 are classified as pro-inflammatory cytokines [39]. Inflammatory factors can inhibit hematopoiesis by destroying hematopoietic stem and progenitor cells, ultimately leading to bone marrow damage [40]. SZEK regulates cytokine levels and attenuates inflammation. The tissue-protective and pro-hematopoietic effects of SZEK may be related to the regulation of inflammation.

The relationship between human health and intestinal flora is highly correlated, with the ability of intestinal flora to influence inflammation and hematopoiesis [15, 41]. In the present study, the intestinal flora of mice with hematopoietic disorders changed considerably after SZEK administration. At the phylum level, SZEK administration decreased the levels of Firmicutes, Proteobacteria, and Actinobacteria, and increased the levels of Bacteroidetes and Verrucomicrobia in the intestines of mice with hematopoietic dysfunction. Firmicutes, Proteobacteria, and Actinobacteria had pro-inflammatory effects, whereas Bacteroidetes and Verrucomicrobia had anti-inflammatory effects [42, 20], confirming that SZEK inhibited inflammation by regulating intestinal flora. The downregulation of inflammation was in line with above ELISA results. LEfSe analysis revealed that genus *Akkermansia* was one of the dominant nodal bacteria in the SZEK group. This genus can activate myelopoiesis and regulate hematopoietic homeostasis [43], demonstrating that the pro-hematopoietic effects of SZEK may be modulated by intestinal flora. At the genus level, SZEK increased the abundance of the probiotic bacterium *Prevotella* in the intestinal tracts of mice with hematopoietic dysfunction. *Prevotella* improves hematopoiesis through cellular immunomodulation [44], suggesting that SZEK promotes hematopoiesis by increasing the levels of beneficial hematopoietic bacteria. Additionally, further analysis of the genus-level flora revealed that *Parabacteroides*, *Bacteroides*, *Helicobacter, Faecalibacterium*, *Streptococcus*, *Akkermansia,* and *Prevotella* functioned in association with macrophages. *Parabacteroides*-derived microbial metabolites promote macrophage polarization toward the M2 type [45], while *Bacteroides* enhance the phagocytosis of macrophages polarized to the M1 type [46]. Low multiplicity of infection of *Helicobacter* promotes M1 and M2 macrophage, whereas high multiplicity of infection suppresses the M2 type [47]. *Faecalibacterium* and *Streptococcus* inhibits the production of pro-inflammatory cytokines by macrophages [48, 49]. *Akkermansia* enhances the anti-inflammatory type of macrophages [50], and *Prevotella* treatment reduces the macrophage M1/M2 ratio [51]. These results suggest that SZEK regulates hematopoiesis through the intestinal flora, which may be associated with macrophage differentiation.

The intestinal flora plays an important role in regulating inflammation and host metabolism, including those of short-chain fatty acids and tryptophan metabolites [52]. Metabolomics could be a predictive tool for deciphering inflammatory pathways [53]. SZEK administration increased stachydrine levels in mice with hematopoietic dysfunction. stachydrine promotes hematopoietic cell production [54], suggesting that SZEK promotes hematopoiesis by regulating serum metabolites. Comprehensive metabolomic analysis facilitated the identification of oxidative phosphorylation as well as valine, leucine, and isoleucine biosynthesis metabolic pathways involved in the enrichment. Oxidative phosphorylation generates energy during HSC activation to meet metabolic requirements [55]. The amino acid Valine acts as a potent enhancer of HSCs, facilitating the maintenance of their self-renewal capacity and promoting the proliferation of HPCs [56]. Combined analysis of intestinal flora and metabolomics revealed a positive correlation between the bacteria *Akkermansia* and *Prevotella*, which can promote hematopoiesis, and metabolites that promote hematopoiesis, such as stachydrine. This was consistent with the previously discussed functions of the intestinal flora and metabolites, suggesting that SZEK can mitigate hematopoietic dysfunction in mice by synergistically regulating the intestinal flora and serum metabolites. Further analyses revealed that the functions of some metabolites were associated with macrophages. Nepodin stimulates AMPK phosphorylation [57], which promotes the transition of macrophages from M1 to M2 [58]. Additionally, 3-HBA produces acetyl coenzyme A [59], which enhances aerobic glycolysis in lipopolysaccharide-activated macrophages to support inflammatory responses [60]. L-carnitine directly inhibits macrophage activation [61]. Correlation analysis of the metabolites further demonstrated a positive correlation between nepodin and 3-HBA, which synergistically promoted macrophage polarization. Consistent with the intestinal flora, metabolomics revealed that the hematopoietic regulatory mechanism of SZEK was associated with macrophages.

Macrophages are among the most important cells and are essential for hematopoietic regulation [6]. Cytokines secreted by macrophages control the development of HPCs into myeloid, lymphoid, and erythroid lineages by stimulating cell cycle progression, proliferation, differentiation, and apoptosis [62]. IL-10 and TGF-β are M2 macrophage-associated factors [27]. IL-10 levels are elevated in hematopoietic mice and inhibit HPCs expansion [8, 63]. TGF-β slows cell cycle progression in HSCs and inhibits their self-renewal potential [7]. SZEK administration reduced the levels of the M2 macrophage surface marker, CD206, in the bone marrow and spleen of hematopoietically dysfunctional mice, suggesting that SZEK administration inhibited M2 macrophage polarization. SZEK administration also inhibited the expression of hematopoietic inhibitory factors such as IL-10 and TGF-β in the spleen of hematopoietically dysfunctional mice. These results suggest that SZEK improves hematopoiesis by inhibiting the polarization of macrophages toward the M2 type and suppressing the production of negative hematopoietic regulatory factors.

This study had some limitations, such as the failure to identify the specific active substances on which SZEK acts. Furthermore, changes in the intestinal flora and metabolites are complex, and whether other mechanisms associated with M2 macrophages exist remains to be explored.

## CONCLUSIONS

In conclusion, our mouse model of hematopoietic dysfunction established using CTX confirmed that SZEK improved bone marrow and spleen pathology, increased the number of hematopoietic cells in the bone marrow, attenuated inflammation, and had a pro-hematopoietic effect. The mechanism involved the modulation of intestinal flora and serum metabolites, which in turn inhibited the differentiation of M2 macrophages and secretion of hematopoietic inhibitory factors.

## MATERIALS AND METHODS

### Animal experimental protocols

This study was performed in accordance with the regulations of the Institutional Animal Ethics Committee of Jilin University (SY202009001). Forty-five 7–8-week-old male SPF BALB/C mice (20 ± 2 g, SY202207012) were purchased from Liaoning Changsheng Biotechnology Co., Ltd. (Liaoning, China). They were housed in a controlled environment at a temperature of 24 ± 2° C, under a 12/12 h light/dark cycle. They had ad libitum access to food and water. CTX (Sigma Aldrich, St. Louis, MO, USA) used in this study was dissolved in saline.

After one week of acclimatization, 27 mice were randomly selected and injected intraperitoneally with 100 mg/kg CTX for 3 days to establish a mouse model of hematopoietic dysfunction, and 75 mg/kg CTX was injected intraperitoneally every 7 days to maintain the stability of the model. The mice were randomly divided into three groups: a model group (Model), an SZEK administration group (SZEK; Jilin Changbaishan Pharmaceutical Group Co., Ltd., Jilin, China) and a rhG-CSF administration group (rhG-CSF, Qilu Pharmaceutical Co., Ltd., Jinan, China). The SZEK group was gavaged with 0.3 ml/25 g of SZEK per day, the rhG-CSF group was injected intraperitoneally with 22.5 ug/kg of rhG-CSF, and the Model group was gavaged with 0.2 mL/20 g of saline per day for 43 days. The remaining 18 mice were injected intraperitoneally with 0.2 mL/20 g) for 3 days. The mice were randomly divided into two groups, gavaged with 0.2 mL/20 g saline (Ctrl) and 0.3 mL/25 g SZEK for the following 43 days.

Two hours after the last treatment, blood samples were obtained from the mice from the caudal vein. After euthanasia, the heart, liver, spleen, kidney, and lung tissues were collected from all groups of mice, and the femur, tibia, and cecum contents were collected in a sterile environment. The collected samples were stored at -80° C.

### Flow cytometry assay

Bone marrow cells were extracted from the femurs and tibiae in a sterile environment. Erythrocytes were removed from the above cells according to the manufacturer’s instructions for the RBC lysis buffer (00-4300-54; Gibco BRL, CA, USA), and the concentration of cells was adjusted to 10^6^ cells/100 μL using staining buffer and staining was performed. The surface markers of the cell populations to be stained were as follows, depending on the cell surface markers: naïve cells (Lin^−^), HSCs (Lin^−^Sca-1^+^c-Kit^+^), LT-HSCs (Lin^−^Sca-1^+^c-Kit^+^CD48^−^CD150^+^), B lymphocytes (CD45^+^CD19^+^) and macrophages (CD11b^+^F4/80^+^). The antibodies utilized for staining, along with their corresponding isotype control antibodies, are presented in Supplementary Table 5. The experimental protocols were performed according to the manufacturer’s instructions. After staining, bone marrow cells were analyzed by flow cytometry in a light-free environment.

### ELISA

The collected blood was allowed to settle and centrifuged at 3,500 rpm for 5 min, and the supernatant was separated. After centrifugation under the same conditions, the supernatant was aspirated as the serum for testing. Subsequently, 100 μL saline was added to 10 mg of frozen spleen tissue, after which the tissue was transferred to a high-throughput tissue grinder and crushed completely. Centrifugation was then carried out at a speed of 12,000 rpm for 5 min, followed by the collection of the supernatant and subsequent centrifugation under identical conditions.

After detecting the protein content in the homogenate according to the BCA kit instructions, the expression levels of IL-4, IL-6, IFN-γ, and TNF-α in the serum and spleen homogenate of hematopoietically dysfunctional mice were determined according to the manufacturer’s instructions. Information on the reagent kits used is shown in Supplementary Table 6.

### Intestinal flora analysis

Four samples of cecum content were collected from each of the Ctrl, Model, and SZEK groups for intestinal flora analysis. Total DNA was extracted from the samples using an OMEGA Soil DNA Kit (M5635-02; OMEGA Bio-Tek, GA, USA) and the samples were quantified using a PicoGreen dsDNA Detection Kit (P7589; Invitrogen, CA, USA). After sequencing, OTU diversity analysis was performed to compare differences in the abundance of intestinal flora among the three groups of mice [64]. The bacterial sequences were uploaded to the NCBI Sequence Read Archive under the accession number PRJNA1008448 (https://www.ncbi.nlm.nih.gov/sra/PRJNA1008448).

### Serum metabolomics analysis

Four serum samples were collected from the Ctrl, Model, and SZEK groups for metabolomic analysis. Serum samples were upsampled on an ultra-performance liquid chromatography (UPLC) system (Agilent 1290 Infinity LC, Thermo Fisher Scientific, Waltham, MA, USA) configured with an ACQUITY UPLC BEH Amide chromatography column (2.1 mm × 100 mm). Serum was analyzed for significant changes in differential metabolites using chromatography-mass spectrometry, and the differential metabolites were subjected to cluster and KEGG analyses, as in our previous study [65].

### H&E staining analysis

The femurs were fixed in 4% paraformaldehyde (BL539A; Biosharp, Guangzhou, China) for 48 h and decalcified in an EDTA decalcification solution. The heart, liver, spleen, kidney, and lung of mice were similarly fixed in 4% paraformaldehyde for 48 h, and along with the decalcified femurs, were dehydrated with gradient alcohol, dipped in wax, and embedded to obtain tissue wax blocks.

The paraffin sections were deparaffinized in water, stained with hematoxylin for nuclear staining and eosin for cytoplasmic staining, dehydrated, sealed with neutral gum, and observed and analyzed under a microscope [64].

### Immunofluorescence analysis

The paraffin sections of decalcified femurs were dewaxed in water for antigen repair, trans-sectioned into 3% hydrogen peroxide solution, incubated at 22° C, protected from light for 25 min, closed with 10% goat serum, incubated overnight with primary antibody (CD206), washed, incubated with secondary antibody for 50 min, and stained with tyramine signal amplification reagent. Subsequently, the nuclei of the cells were re-stained with 4',6-diamidino-2-phenylindole, sealed, and observed and analyzed under a microscope [66]. Information on the antibodies used is shown in Supplementary Table 7.

### Western blotting

The spleen tissues were mixed with RIPA lysate (20-188; Millipore, MA, USA) containing a cocktail of protease and phosphatase inhibitors (P002; NCM Biotech, Jiangsu, China), ground in a high-throughput tissue mill, and centrifuged at 12,000 rpm for 5 min. Subsequently, the supernatant was extracted and centrifuged again under the same conditions, and the supernatant obtained from the final separation was the spleen lysate. The total protein content of the spleen lysate was measured according to the BCA kit instructions (A53225; Thermo Fisher Scientific, MA, USA).

Proteins were separated using 10–12.5% SDS-PAGE gels at 90–120 V. When proteins with different molecular weights were completely separated, they were transferred to polyvinylidene difluoride membranes. The membranes were sealed with a rapid closure solution (GF1815; GeneFirst, Shanghai, China) and incubated overnight with primary antibodies specific to the target proteins (CD206, ARG1, IL-10, TGF-β, GAPDH). After washing, they were incubated again with the corresponding secondary antibody. Target protein expression was detected using a highly sensitive chemiluminescence detection kit (GK10008; GlpBio, CA, USA) and a gel imager (Tanon 5200; Tanon Science and Technology, Shanghai, China) [65]. ImageJ v1.8.0 (National Institutes of Health, Bethesda, MD, USA) was used for quantitative analysis. Information on the antibodies used is presented in Supplementary Table 8.

### Statistical analysis

The results of this study are expressed as mean ± standard deviation. Data were analyzed using BONC DSS Statistics v25.0 (IBM Corporation, Armonk, NY, USA), and one-way analysis of variance and Tukey’s post hoc multiple ratios were used to analyze the statistical significance between groups. The groups were considered significantly different when *P*-value < 0.05.

## Supplementary Material

Supplementary Tables
