# Supplementary material for: Impact of macrophage differentiation on hematopoietic function enhancement by Shenzhu ErKang Syrup
Source: Aging (Albany NY). 2024 Jan 3;16(1):169–90. doi: 10.18632/aging.205358 (PMC10817372; doi:10.18632/aging.205358)
Supplement: Supplementary Tables [file aging-16-205358-s001.pdf]

SUPPLEMENTARY TABLES

Supplementary Table 1. Statistics on the number of phylum levels microbial taxonomic units.

Name	Ctrl	CTX (100 mg/kg)	
		Model	SZEK (0.3 mL/25 g)
Firmicutes	0.46157667	0.51315607	0.40695913
Bacteroidetes	0.43584508	0.34426736	0.43881115
Proteobacteria	0.06246042	0.09866328	0.05411347
Verrucomicrobia	0.00004259	0	0.07475021
Tenericutes	0.00651937	0.00393053	0.00959245
Deferribacteres	0.00581225	0.00713290	0.00479138
Actinobacteria	0.00308173	0.00481343	0.00100596
Cyanobacteria	0.00022432	0.00010390	0.00004693
Others	0.02443759	0.02793254	0.00992931

CTX, cyclophosphamide; Ctrl, control; Model, hematopoietic dysfunction model; SZEK, Shenzhu Erkang Syrup. Data are presented as the mean.

Supplementary Table 2. Relative abundance of top 20 genera.

Name	Ctrl	CTX (100 mg/kg)	
		Model	SZEK (0.3 mL/25 g)
Muribaculum	0.263865114	0.196806737	0.217482401
Lactobacillus	0.053710125	0.078176736	0.078849051
Lachnoclostridium	0.067121873	0.063661338	0.015631407
Bacteroides	0.029019545	0.055069626	0.048742486
Desulfovibrio	0.042771832	0.027256729	0.021612370
Alistipes	0.030890482	0.022080630	0.022161949
Akkermansia	0.000042589	0	0.074229185
Escherichia	0.000042992	0.060558506	0.003708398
Eisenbergiella	0.003250962	0.023201912	0.010672943
Ruminococcus	0.012312047	0.011173167	0.007562315
Helicobacter	0.006231553	0.002897073	0.016474879
Mucispirillum	0.005812247	0.007132897	0.004791381
Anaerotruncus	0.003313994	0.005934737	0.008125723
Prevotella	0.001396223	0.004973241	0.008639600
Parabacteroides	0.006610887	0.002601051	0.004190403
Kineothrix	0.002487264	0.004829357	0.006033109
Erysipelatoclostridium	0.005797538	0.004210397	0.001740779
Odoribacter	0.001538028	0.001779274	0.003193652
Faecalibacterium	0.002665859	0.000744821	0.000966145
Streptococcus	0.000809606	0.001463203	0.000532411

CTX, cyclophosphamide; Ctrl, control; Model, hematopoietic dysfunction model; SZEK, Shenzhu Erkang Syrup. Data are presented as the mean.

Supplementary Table 3. The dominant taxa of intestinal flora among Ctrl, model and SZEK.

Group	Dominant taxa	Abundance (log_{10})	LDA score (log_{10})	*P*-value
Ctrl	Bacteria. Tenericutes. Mollicutes. Entomoplasmatales	3.663142625	3.672016886	0.024439894
	Bacteria. Bacteroidetes. Bacteroidia. Bacteroidales. Muribaculaceae	5.421504681	4.574632518	0.049787068
	Bacteria. Tenericutes. Mollicutes. Entomoplasmatales. Spiroplasmataceae	3.663142625	3.607057354	0.024439894
	Bacteria. Bacteroidetes. Bacteroidia. Bacteroidales. Muribaculaceae. Muribaculum	5.421381631	4.519135488	0.049787068
Model	Bacteria. Actinobacteria. Coriobacteriia. Eggerthellales. Eggerthellaceae	3.678545647	3.515615699	0.030783722
	Bacteria. Bacteroidetes. Bacteroidia. Bacteroidales. Rikenellaceae. Alistipes. Alistipes_shahii	2.993761609	4.082646265	0.037311207
	Bacteria. Actinobacteria. Coriobacteriia. Eggerthellales	3.678545647	3.532011882	0.030783722
	Bacteria. Actinobacteria. Coriobacteriia	3.682454909	3.561022123	0.030783722
	Bacteria. Actinobacteria	3.682454909	3.568636281	0.030783722
	Bacteria. Firmicutes. Erysipelotrichia. Erysipelotrichales. Erysipelotrichaceae. Erysipelatoclostridium. _Clostridium__cocleatum	2.998480681	3.668499118	0.047860072
SZEK	Bacteria. Verrucomicrobia	4.873612894	4.656633937	0.009214688
	Bacteria. Verrucomicrobia. Verrucomicrobiae. Verrucomicrobiales. Akkermansiaceae	4.873612894	4.654910595	0.009214688
	Bacteria. Firmicutes. Clostridia. Clostridiales. Clostridiaceae. Butyricicoccus	2.798752648	4.263618902	0.044060991
	Bacteria. Verrucomicrobia. Verrucomicrobiae	4.873612894	4.643335534	0.009214688
	Bacteria. Tenericutes. Mollicutes. Acholeplasmatales. Acholeplasmataceae	3.822916478	3.557580482	0.011927663
	Bacteria. Verrucomicrobia. Verrucomicrobiae. Verrucomicrobiales. Akkermansiaceae. Akkermansia	4.870575179	4.666489858	0.009214688
	Bacteria. Bacteroidetes. Bacteroidia. Bacteroidales. Bacteroidaceae. Bacteroides. Bacteroides_vulgatus	3.418878172	3.598089591	0.028541647
	Bacteria. Verrucomicrobia. Verrucomicrobiae. Verrucomicrobiales. Akkermansiaceae. Akkermansia. Akkermansia_muciniphila	4.851747599	4.623122189	0.005365730
	Bacteria. Verrucomicrobia. Verrucomicrobiae. Verrucomicrobiales	4.873612894	4.672322833	0.009214688
	Bacteria. Tenericutes. Mollicutes. Acholeplasmatales	3.822916478	3.534082167	0.011927663

The current LDA threshold is 2. Differences were considered statistically significant at $P < 0.05$. LDA, Linear Discriminant Analysis; Ctrl, control; Model, hematopoietic dysfunction model; SZEK, Shenzhu Erkang Syrup. Data are presented as the mean.

Supplementary Table 4. Differential metabolites with significant changes between groups in serum metabolomics.

Name	Ctrl	CTX (100 mg/kg)		CTX vs Ctrl		SZEK vs CTX	
		Model	SZEK (0.3 mL/25 g)	VIP	*P*-value	VIP	*P*-value
3-hydroxybutyric acid	43511826.35	110913649.40	81559201.98	10.38061467	0.045996402	18.31680887	0.000093855
3-methyl-l-histidine	65623280.92	47451297.83	68984919.95	1.720657984	0.045529809	1.629884797	0.042358862
D-Quinovose	170245777.80	136014120.40	326330944.10	2.324055497	0.021646538	5.248964999	0.000026258
Eicosenoic acid	619536917.40	436898215.10	706617874.90	5.327792258	0.022136843	5.859911076	0.008482386
Isobutyric acid	60559608.19	124289215.00	64481854.22	3.230295093	0.038398413	2.664879395	0.049272208
L-carnitine	8966428635.00	7827757011.00	10496250901.00	12.74641862	0.000048872	18.30632963	0.000631104
L-pipecolic acid	117537126.30	73807406.29	124829071.80	2.478225091	0.023324898	2.633984682	0.000915433
L-threonate	183304124.30	144985774.90	402950879.20	2.097759706	0.038843211	6.108588501	0.000226973
Pe 34:0	13556965.14	5496519.96	13198999.42	1.188473083	0.004244829	1.010415125	0.001094283
Sarcosine	244461117.80	210570116.20	280977434.20	2.211526814	0.039664048	3.032502501	0.003045672
Stachydrine	349304065.50	214497719.70	768032150.00	3.983220667	0.041417312	8.449337388	0.000676251
Tetraethylene glycol	456647994.70	980550760.80	460080407.30	9.269579520	0.037299241	7.959096532	0.036819691
Trans-2-hydroxycinnamic acid	69661984.24	56809262.09	133625195.80	1.439023999	0.012052976	3.333455519	0.000010032
Triethylene glycol monobutyl ether	35863220.06	104612687.70	34916458.12	3.371029305	0.032969338	2.918171761	0.030694915
Nepodin	204045291.90	283118064.30	45519511.03	3.503461489	0.023327089	5.951019208	0.000037633

CTX, cyclophosphamide; Ctrl, control; Model, hematopoietic dysfunction model; SZEK, Shenzhu Erkang Syrup. Data are presented as the mean.

Supplementary Table 5. The antibody information of flow cytometry.

Name	Item number	Manufacturer
FITC anti-mouse Lineage Cocktail with Isotype Ctrl	133302	Biolegend
PE anti-mouse Ly-6A/E (Sca-1) Antibody	108108	Biolegend
APC/Cyanine7 anti-mouse CD117 (c-kit) Antibody	105826	Biolegend
APC anti-mouse CD48 Antibody	103411	Biolegend
Brilliant Violet 421™ anti-mouse CD150 (SLAM) Antibody	115925	Biolegend
APC anti-mouse CD45 Antibody	147707	Biolegend
PE anti-mouse CD19 Antibody	115507	Biolegend
FITC anti-mouse/human CD11b Antibody	101205	Biolegend
APC anti-mouse F4/80 Antibody	123116	Biolegend
APC Armenian Hamster IgG Isotype Ctrl	400912	Biolegend
PE Rat IgG2a, κ Isotype Ctrl	400507	Biolegend
APC/Cyanine7 Rat IgG2b, κ Isotype Ctrl	400624	Biolegend
Brilliant Violet 421™ Rat IgG2a, κ Isotype Ctrl	400549	Biolegend
APC Rat IgG2b, κ Isotype Ctrl	400611	Biolegend
FITC Rat IgG2b, κ Isotype Ctrl	400605	Biolegend
APC Rat IgG2a, κ Isotype Ctrl	400511	Biolegend

Supplementary Table 6. The information of reagent kits.

Name	Item number	Manufacturer
Pierce BCA Protein Assay Kits	A53225	Thermo Fisher
Mouse IL-4 ELISA Kit	ml002149-1	mlbio
Mouse TNF-α ELISA Kit	ml002095-1	mlbio
Mouse IL-6 ELISA Kit	ml063159-1	mlbio
Mouse IFN-γ ELISA Kit	ml002277-1	mlbio

Supplementary Table 7. The antibody information of immunofluorescence staining.

Name	Item number	Manufacturer	Dilution rate
CD206	18704-1-AP	Proteintech	1:200
Anti-Rabbit IgG (H+L) Antibody	5220-0336	SeraCare	1:400

Supplementary Table 8. The antibody information of Western blotting.

Name	Item number	Manufacturer	Molecular weight (kDa)	Dilution rate
CD206	18704-1-AP	Proteintech	170	1:1000
ARG1	A1847	ABclonal	27	1:2000
IL-10	A2171	ABclonal	18	1:2000
TGF-β	A15103	ABclonal	44	1:2000
GAPDH	E-AB-48016	Elabscience	37	1:1000
Goat Anti-Rabbit (H+L)	E-AB-1003	Elabscience	—	1:2000
Goat-Anti-Mouse (H+L)	E-AB-1001	Elabscience	—	1:2000
